# Rubella immunity and serum perfluoroalkyl substances: Sex and analytic strategy

**DOI:** 10.1371/journal.pone.0203330

**Published:** 2018-09-24

**Authors:** Courtney S. Pilkerton, Gerald R. Hobbs, Christa Lilly, Sarah S. Knox

**Affiliations:** 1 WVU School of Medicine, West Virginia University, Morgantown, West Virginia, United States of America; 2 Deptarment of Statistics, West Virginia University, Morgantown, West Virginia, United States of America; 3 Department of Biostatistics, School of Public Health, West Virginia University, Morgantown, West Virginia, United States of America; 4 Department of Epidemiology, School of Public Health, West Virginia University, Morgantown, West Virginia, United States of America; Stony Brook University, Graduate Program in Public Health, UNITED STATES

## Abstract

**Background:**

Perfluoroalkyl substances (PFASs) have been associated with decreased immunity to childhood tetanus and diphtheria immunizations. If these vaccinations are vulnerable to influence from PFASs, questions arise about associations with other common inoculations.

**Objective:**

To examine whether serum PFASs were associated with reduced immunity to rubella immunization, and whether interactions with sex or ethnicity warranted analytic stratification. Usually, toxicology analyses are calculated controlling for race and sex. However, sex differences in immune function have been reported and a reduction of immunity to rubella in women could pose risks such miscarriage.

**Methods:**

We analyzed a nationally representative sample of individuals ≥ 12 years from the National Health and Nutrition Examination Survey (NHANES) for years 1999–2000 and 2003–2004 for whom PFAS measures were available. Our analytic strategy was to start with separate analyses for youth and adults controlling for several covariates including ethnicity and sex, as well as the interaction of these terms with PFASs. If there was a main effect of PFASs and an interaction term, we would stratify analyses of effect size. The outcome variable was Rubella IgG titers by quartile of perfluoroalkyl substances.

**Results:**

After exclusion for missing data, the analyzed sample contained 581 adult women, 621 adult men, and 1012 youth. There was no significant effect of PFASs on immunity in youths but a significant effect of both PFOA and PFOS in adults, as well as a significant interaction of PFOA x sex and a borderline significant interaction of PFOS x sex. When effect size analyses were stratified by sex, a significant association between rubella titres and PFOA was found in men but not women and PFOS was not significant in either sex.

**Conclusions:**

These results support our earlier studies showing sex specific responses to PFASs and indicate the importance of thinking carefully about analytic strategies in population based toxicology research.

## Introduction

Perfluoroalkyl substances (PFASs), which include perfluorooctanoic acid (PFOA) and perfluorooctane sulfonate (PFOS), are synthetic compounds used in fluoropolymer synthesis. These compounds are important in the manufacture of many fire resistant, stain resistant, and non-stick products [1]. Concern about human exposure to these chemicals has increased due to findings of their ubiquity and persistence in the environment [2–4] as well as their presence in detectable amounts in over 98% of blood samples from a representative sample of the U. S. population [5–6]. PFASs can be ingested from food and contaminated water [7], and seem to be transferred through the placenta during pregnancy [8–9]. Exposure to these chemicals has been linked to liver, lung, and kidney damage as well as to immune changes in rats [10] and mice, [11–13] at concentrations similar to those found in humans [14]. In vitro studies using human cell lines have also shown that PFASs suppress natural killer cell activity and cytokine release [15–16]. More importantly, recent studies in humans suggest an association between PFAS exposure and decreased immune response to vaccination. In a prospective cohort study of children ages 5 and 7 years, it was reported that increased exposure to PFASs was associated with decreased humoral immune response to childhood tetanus and diphtheria immunizations [17]. Another prospective cohort study found that increasing maternal PFAS levels were associated with decreased rubella antibody titers in their children at age 3 [18]. In an adult cohort from a PFOA contamination area it was found that increased PFOA exposure was associated with reduced immune response to influenza vaccination and an increased risk of not meeting the antibody threshold needed for long-term protection [19].

Despite these data, the mechanism through which PFASs might modulate the immune system are still unknown, although there are suggestions of an association with the peroxisome proliferator activated receptor alpha (PPAR alpha) [20]. It is also not known whether other childhood immunizations may be affected by PFAS exposure or whether immune responses in a typical adult are affected. *The objective of this paper* was to test whether the rubella titres of MMR (measles-mumps-rubella) immunization are vulnerable to influence from PFAS exposure in adults and youth. To do this we analyzed NHANES data, a nationally representative sample that measured not only PFASs but also rubella titres. Rubella seemed an important outcome to measure because it causes ‘3-day measles’, which can have the devastating consequence of miscarriage in pregnant women. The relevance of this question is particularly important to pregnant women who have been vaccinated and believe themselves to be resistant [21].

Traditionally, toxicology analyses are very often calculated by controlling for the effect of ethnicity and sex, despite the lack of any clear justification for doing this. The authors have found sex specific physiological responses associated with PFASs in their earlier work [2] but have not examined ethnicity differences. Based on previously published data showing lower PFAS concentrations in menstruating women [22–26] that may be as great as 30% or more [25–26], the hypothesis of this paper was that sex is an effect modifier and that by not stratifying analyses, there is a risk that overall results will mask true associations. Given the importance that a reduction of immunity to rubella in women might pose, this seemed especially important to test.

## Materials and methods

### Participants

We analyzed merged data from the 1999–2000 and 2003–2004 cycles of the National Health and Nutrition Examination Survey (NHANES). In brief, the NHANES survey includes a stratified, cross-sectional, multistage probability sample representative of the civilian non-institutionalized U.S. population. The sample is structured so that in principle, everyone in the U.S. has an equal probability of being selected, and there is oversampling of low-income persons, those 60 years of age or older, African Americans, and Mexican Americans to provide stable estimates for these groups. These particular survey years were chosen because the survey included biomonitoring for PFASs by the National Center for Environmental Health in a random subsample of a third of the participants.

Detailed descriptions of the NHANES study design and methods are described elsewhere [27]. Because the NHANES cohort changes with every iteration, these data are cross-sectional, not prospective. Subjects were required to sign a consent form before participation, and approval was obtained from the Human Subjects Committee of the US Department of Health and Human Services. The data analyzed in this study were anonymized public use data available on the NHANES website.

Our sample consisted of NHANES participants 12 years of age or older for whom measurements were available. There were 1387 adults (19–49), of whom 1202 had data available for PFASs, rubella, and ethnicity and were not pregnant at the time of measurement (87%). Data on young children was not available. Youth were defined as the age group ranging from 12 to 18 years. The resulting participants were 621 adult men, 581 adult women and 1012 youth. Race / ethnicity was a designated classification in the NHANES dataset.

### Rubella IgG titers

In NHANES 1999–2000 and 2003–2004 blood specimens were processed, stored and shipped to Viral and Rickettsial Disease Laboratory, California State Department of Health Services, Berkeley, California for analysis. The laboratory procedures for determining rubella IgG titer are outlined in the Laboratory Procedure Manual: Measles, Rubella, and Varicella-Zoster Antibodies [27]. Rubella titer values were log-transformed due to the skewness of their distribution. Rubella was also classified as protective or non-protective titer; a rubella titer of ≥10 IU is considered protective [28]. Preliminary analyses showed that most of the participants were above this threshold (e.g., 94% of adults and 90% of youth), Seropositivity is a measure of clinical risk but is not a proxy for whether or not someone has been vaccinated [28]. We were concerned that excluding anyone under this threshold might bias our analyses by excluding those individuals most affected by the endocrine disruption of PFASs. Therefore, all of whom were included in the analyses.

### PFOS and PFOA

Rigorous procedures and quality-control were used in blood collection and details about these procedures are available in the NHANES Laboratory Procedures Manual [27]. PFOS and PFOA were measured in serum of participants by the National Center for Environmental Health, using automated solid-phase extraction coupled with isotope dilution high-performance liquid chromatography-tandem mass spectrometry. Details of laboratory methods have been published previously [5]. The limits of detection were 0.1 ng/mL for PFOA and 0.2 ng/mL for PFOS, and the interassay coefficients of variation were 11% for PFOA and 13% for PFOS.

### Covariates

Age, race/ethnicity, and educational level (high school, less than high school, more than high school) were assessed using the demographic questionnaire. BMI was calculated as weight in kilograms divided by height in meters squared. This covariate was included because earlier work summarized by Knox et al. [2] showed that PFOA may vary in obese subjects, leading to their analyzing the data in that paper by BMI. They found inconsistent results and we believe that including it as a covariate was warranted. Because literature suggests that pregnancy and birth impact PFASs levels, such that the higher the number of births the lower the mothers’ PFAS concentration [8], parity was assessed using the reproductive health questionnaire and controlled for in the analyses in women. For these analyses, parity was defined in the dataset as the number of live births. Although stillbirths might also have resulted in loss of PFASs, that data was not available. This was also warranted because the dichotomous variable of having or not having a child in this particular sample is associated with rubella titer. In women, the number of live births was included as a covariate because it was associated with PFAS levels (women lose PFASs with blood). Education was not included as a covariate in the youth analyses because it is linearly associated with age.

### Statistical approach

Earlier research has reported sex differences in the biological effects of PFASs [2], as well as sex [29] and racial [30] differences in humoral immunity. Our earlier research [2, 8] and that of others indicates that circulating PFAS levels in pregnant women and women who have given birth are lower than those of non-pregnant women who have never given birth. The probable reason is that these chemicals, which are otherwise bioacumulative, are transferred to the fetus during gestation [8]. Women also lose PFASs with menstrual blood. However, this does not mean that the association with Rubella titres is influenced by sex. Given earlier significant results of PFASs on serum vaccine antibodies in children [17], we believed that the most logical analytic strategy would be to start by calculating separate analyses in youth and adults. An additional reason is that adult women differ from youth in ways that influence PFAS concentrations. In both age groups (12–18, and ≥ 19–60), we calculated the main effect of PFASs on Rubella titres controlling for the covariates, age, BMI, educational level, sex, ethnicity and the interactions of sex and ethnicity with PFASs. If no significant main effect of PFASs on Rubella titres was found, we would report negative results and not massage the data by doing further analyses. The point of including interaction terms (with sex and ethnicity) was to investigate whether stratified analyses were justified. Earlier studies on PFASs from our group [1, 2, 31] have found sex specific responses to PFASs to be the rule rather than the exception. If significant main effects of PFASs were found, and there were significant interactions of sex or ethnicity with PFASs, we would analyze the data further, stratifying according to the interaction that was significant. The goal was not to compare men to women or ethnicities to one another, but to assess the effect of PFASs on Rubella within group to assure that highly significant effects in one sex /ethnicity group had not generalized to all. Quartiles of PFASs were calculated separately in adults and youth.

Preliminary analyses of the current NHANES dataset, indicated that the ‘yes’ / ‘no’ response to whether or not the respondent had children was significantly associated with rubella titer and that the continuous variable (number of children) was significantly associated with PFOA concentrations. For these reasons and because of the transfer of PFASs to the fetus during pregnancy indicated by earlier work from our group [8], we also made the decision to control for parity in women, should stratification by sex be indicated.

Thus, in the initial analyses we simply fitted multivariable linear regression models separately for the effect of PFOA and PFOS quartiles (treated as categorical variables) of rubella titres in adults and youth, adjusting for age, BMI, ethnicity, education, as well as interactions of sex and ethnicity with PFASs. These analyses were essentially preliminary analyses to investigate whether there was any effect of PFASs on rubella titres and to see whether tests for effect size should be stratified by either sex or ethnicity. Sex had a binary code (0, 1) so a significance in this variable indicated an effect of sex. Analyses were calculated with Proc Surveyreg, SAS version 9.3 to account for survey weights and design (SAS Institute, Inc., Cary, North Carolina).

## Results

### Descriptive statistics

Table 1 presents the characteristics of the study population. Approximately 37% of the sample were non-Hispanic white men, 6% non-Hispanic black men, and 9% Hispanic. Percentages were similar in women. About twenty-three percent of the youth in this cohort were non-Hispanic white, 31% non-Hispanic black, and 42% Hispanic. About 49% were female and 44% of adults lived in households where the highest level of education was more than a high school education. The average PFOA concentration was about 6 ng/mL (standard error ± 0.3) in men, 4.3 ng/mL in women and 4.8 ng/mL in youth. Sex differences in adults were significant. The average PFOS concentration in men was 28.1 ng/mL (standard error ± 0.7), 22.1 ng/mL in women and 25.1 in youth.

**Table 1:** Descriptive statistics (rounded to the nearest decimal)*.

	Men	Pr.	Women	Youth (12–18)
Age (mean ± SE)	34.3 ± 0.7	.0001	35.2 ± 0.7	15.0 ± 0.6
BMI (mean ± SE)	28.0 ± 0.3	.0001	28.5 ± 0.5	23.8 ± 0.2
PFOS ng/mL (mean ± SE)	28.1 ± 1.3	.0001	22.1 ± 0.9	25.1 ± 0.4
PFOA ng/mL (mean ± SE)	6.0± 0.3	.0001	4.3 ± 0.2	4.8 ± 0.7
Rubella Titer IU (mean ± SE)	74.7.± 3.4	.001	69.3 ± 2.7	52.8 ± 2.4
**Race/ethnicity**	%	0.44	%	
Non-Hispanic White (%)	36.6		35.1	22.9
Non-Hispanic Black (%)	5.6		6.7	30.9
Mexican American/ Hispanic (%)	8.5		7.5	41.6
**Education**		0.42		
Less Than High School (%)	8.2		8.5	35.1
High School Grad/GED/Equival (%)	15.4		12.7	21.7
More Than High School (%)	27.0		28.3	39.2

*P-value for significance set at 0.05. Analyses separated by sex in adults due to a significant interaction between sex and PFASs

### Whole group analyses—Youth

Whole group linear regression analyses of youth (12–18 years) showed no significant associations with Rubella titres for either PFOA or PFOS adjusting for covariates, nor were there interactions of PFASs with sex or ethnicity. The results can be seen in Table 2. Thus, in adherence with our analytic strategy, we did not stratify or analyze this group any further.

**Table 2:** Youth*.

Effect (N = 1196)	F Value	Pr > F
**PFOA**		
PFOA quartile	0.34	0.7974
Sex	0.31	0.5828
Ethnicity	14.47	<.0001
Sex*PFOA quartile	0.32	0.8088
Ethnic*PFOA quartile	0.68	0.6637
**PFOS**		
PFOS Quartile	1.44	0.2512
Sex	0.27	0.6045
Ethnicity	18.59	<.0001
Sex*PFOS quartile	0.88	0.4631
Ethnic*PFOS quartile	1.33	0.2742

*Regression analyses of log rubella titres on PFOA and PFOS quartiles adjusted for age, sex, educational level, ethnicity, BMI and sex/ethnicity interaction terms.

### Whole group analyses—Adults

In adults, there was a significant association between both PFOA (p = 0.0016) and PFOS (p = 0.0295) quartiles and rubella titers after controlling for covariates (sex, age, ethnicity, education, BMI and the interactions between sex and PFOA and ethnicity and PFOA). Interactions between sex and PFOA were significant (p = 0.0193) but with PFOS only borderline significant (0.0609) in adults. These can be seen in Table 3.

**Table 3:** Adults*.

Effect (N = 1193)	F Value	Pr > F
**PFOA**		
PFOA Quartile	6.60	0.0016
Sex	3.50	0.0716
Ethnicity	4.21	0.0249
Sex * PFOA quartile	3.86	0.0193
Ethnic * PFOA quartile	1.92	0.1117
**PFOS**		
PFOS Quartile	3.44	0.0295
Sex	1.82	0.1883
Ethnicity	3.74	0.0359
Sex * PFOS quartile	2.75	0.0609
Ethnic * PFOS quartile	0.89	0.5179

*Regression analyses of log rubella titres on PFOA and PFOS quartiles adjusted for age, gender, ethnicity, BMI, educational level and sex/ ethnicity interaction terms.

Due to the significant sex x PFOA interaction and borderline significant PFOS interaction, we stratified the subsequent analyses for estimates and confidence intervals by sex, fitting multivariable linear regression models to calculate and compare log rubella IgG titer for each quartile of PFOS and PFOA. The lowest quartile was used as the referent.

### Analyses stratified by sex

There were no significant associations between rubella titres and either PFOA or PFOA concentrations in women. In men, only PFOA showed significant negative associations with rubella titres, indicating that the higher the PFOA, the lower the Rubella titre. The closest PFOS came to significance in men was p = 0.08 between the second and first quartiles. The PFAS data for both men and women can be seen in Table 4.

**Table 4:** Adult analyses stratified by sex.

WOMEN				MEN			
Effect (N = 542)	Estimate	Pr > T	Confidence Intervals	Effect (N = 613)	Estimate	Pr > T	Confidence Intervals
**PFOA** **Quartile**				**PFOA** **Quartile**			
Quartile 1	0.0000			Quartile 1	0.0000		
Quartile 2	-0.2536	0.0636	-0.52, 0.02	Quartile 2	- 0.1382	0.3387	-0.43, 0.15
Quartile 3	-0.1495	0.6856	-0.90, 0.60	Quartile 3	-0.5477	0.0002	-0.81, -0.28
Quartile 4	-0.1658	0.6769	-0.97, 0.64	Quartile 4	-0.4450	0.0278	-0.84, -0.05
**PFOS** **Quartile**				**PFOS** **Quartile**			
Quartile 1	0.000			Quartile 1	0.0000		
Quartile 2	0.0450	0.81	-0.34, 0.43	Quartile 2	-0.1987	0.35	-0.62, 0.23
Quartile 3	0.0439	0.87	-0.51, 0.60	Quartile 3	-0.3179	0.08	-0.69, 0.05
Quartile 4	-0.1664	0.73	-1.13, 0.80	Quartile 4	0.0086	0.97	-0.54, 0.56

Regression analyses of log rubella titres on PFOA and PFOS quartiles adjusted for age, sex, ethnicity, BMI and educational level, as well as number of live births in women (only in women because they lose PFASs with blood during delivery).

## Discussion

When men and women were analyzed together, controlling for sex and ethnicity, both PFOA and PFOS showed significant associations with rubella titre. The interaction for sex x PFOA was highly significant, whereas the interaction of sex with PFOS was only borderline. In analyses stratified by sex, PFOA showed significant negative associations with rubella titres only in men. One quartile of PFOS was also borderline significant in men. This illustrates that a strong value in only one sex can carry over into an overall significance for the whole group when sex is adjusted for in the analyses, leaving the reader with incorrect conclusions about risk. Table 1 shows that the differences in levels of rubella titres between men and women are significant. This apparent discrepancy could be partly due to the previously mentioned sex differences related to menstrual cycles [22–26] and parity. If women have lower concentrations of PFASs to begin with, it may explain why the subsequent stratified analyses controlling for parity in women show a more pronounced effect of PFOA than PFOS on rubella titres in men.

### Previous findings

Unlike previous findings associated with tetanus, diphtheria immunity [17], this study does not show immune system effects of PFASs in youth, however, the children in the earlier study, a Danish birth cohort, were under the age of 5, whereas our study included only youth between 12–18 years. The earlier study found that elevated PFAS exposure was associated with a decreased humoral immune response and increased odds of having an antibody level below a clinically protective level. As we had no children under 5 in our dataset we were unable to test whether that age group might also be especially vulnerable to PFAS effects on rubella immunity. However, a more recent study [32] also analyzed NHANES data, defining youth as ages 12–19 found a significant negative correlation between rubella titre and PFASs in the seropositive group only. It was also unclear to us why youths up to the age of 19 were classified as children in that study and we chose to use the more common cutoff of 18. As to seropositivity, there are multiple possible approaches. We chose not to do separate analyses for seropositive and seronegative groups for the several reasons. Our earlier research and that of others [2, 8–9] has shown that PFASs are endocrine disrupters and accumulating data support an association between endocrine disruption and the immune system [33–35]. There is justification for both approaches and we believe that this supports our contention that careful thought should be given to analytic strategy due to its important consequences for interpretation of results.

### Traditional toxicology analysis strategies

It has been traditional in toxicology analyses to use sex as a covariate in multivariable regression equations [17, 19]. We believe that for a number of reasons this can lead to misleading interpretation of the data. First, a statistically large effect in one sex can generalize to the entire group when analyses are adjusted for sex, making it look as if the effect is generalized across all. Second, men and women are fundamentally different physiologically. This has been demonstrated in our previous work with PFOA and PFOS [2, 8, 29].

The varying effect of PFAS exposure on immunity in men and women found in this study is actually not surprising, not only because women have lower concentrations than men but also because women are known to mount a more robust immune response to antigenic challenge than men. Women have shown increased antibody responses to many vaccines; mumps [36–37], smallpox [38], influenza [39], and rubella [36, 40] when compared with men. With respect to the influenza vaccine women have been found to respond to a half dose of vaccine at comparable levels to men given a full dose [39]. The increased robustness of the immune response in women may explain why they seemed to be less vulnerable to reduced rubella immunity from PFAS exposure than men. It should also be noted that women have higher rates of many autoimmune diseases [41]; scleroderma, Sjogren’s syndrome, rheumatoid arthritis and systemic lupus erythematosus suggesting other sex related differences in immunity Mediating mechanisms such as pharmacokinetics, receptor signaling and effect on immune cells require further research.

### Limitations

The main limitation of this study is that NHANES data is cross-sectional and therefore the temporal nature of the association between PFASs and humoral immune response could not be determined. We also did not have young children in this cohort. There are other factors that might have influenced rubella titres that were not available for examination. There was no information concerning whether the participants had actually been vaccinated or had had rubella infection which could influence seropositivity. There was also no information as to the age at which vaccination occurred, other infections, or variation in vaccination rates and schedules.,

Despite the caveats, we believe that these data indicate caution in the way toxicology data are analyzed and a preference for stratifying by rather than controlling for sex. A clear rationale for analytic strategies should be included in reports.

## Conclusions

Our findings suggest that there are sex specific responses in the immunity to rubella associated with PFAS exposure and that it is important to include examination of sex x exposure interactions when analyzing immune responses to potentially toxic agents.

## References

[1] Frisbee SJ, Shankar A, Knox SS, Steenland K, Savitz DA, Fletcher T,. Perfluorooctanoic acid, perfluorooctanesulfonate, and serum lipids in cildren and adolescents: results from the C8 Health Project. *Arch Pediatr Adoles Med* 2010;164:860–869. 10.1001/archpediatrics.2010.163 PMC3116641 20819969

[2] Knox SS, Jackson T, Frisbee SJ, Javins B, Ducatman AM. Perfluorocarbon exposure, gender and thyroid function in the C8 Health Project. J Toxicol Sci 2011; 36;403–410. 21804304 10.2131/jts.36.403

[3] Lin C-Y, Chen P-C, Lin Y-C, Lin L-Y. Association among serum perfluoroalkyl chemicals, glucose homeostasis, and metabolic syndrome in adolescents and adults. Diabetes Care 2009; 32:702–707. 10.2337/dc08-1816 19114613 PMC2660466

[4] Melzer D, Rice N, Depledge MH, Henley WE, Galloway TS. Association between serum perfluorooctanoic acid (pfoa) and thyroid disease in the us national health and nutrition examination survey. Environ Health Perspect 2010;118:686 10.1289/ehp.0901584 20089479 PMC2866686

[5] Calafat AM, Wong L-Y, Kuklenyik Z, Reidy JA, Needham LL. 2007. Polyfluoroalkyl chemicals in the us population: Data from the national health and nutrition examination survey (nhanes) 2003–2004 and comparisons with nhanes 1999–2000. Environm Health Perspectives 2007:115:1596–1602. 10.1289/ehp.10598 PMC2072821 18007991

[6] Lau C, Anitole K, Hodes C, Lai D, Pfahles-Hutchens A, Seed J. Perfluoroalkyl acids: A review of monitoring and toxicological findings. Toxicol Sci 2007;99:366–394. 10.1093/toxsci/kfm128 17519394

[7] Vestergren R, Cousins IT. 2009 Tracking the pathways of human exposure to perfluorocarboxylates. Environmental science & technology 43:5565–5575. 19731646 10.1021/es900228k

[8] Javins B, Hobbs G, Ducatman AM, Pilkerton C, Tacker D, Knox SS. Circulating maternal perfluoroalkyl substances during pregnancy in the c8 health study. Environ Science & Technol 2013 47:1606–1613. 10.1021/es3028082 23272997

[9] Needham LL, Grandjean P, Heinzow B, Jørgensen PJ, Nielsen F, Patterson DG Jr, et al Partition of environmental chemicals between maternal and fetal blood and tissues. Environ Sci & Technol 2010;45:1121–1126. 21166449 10.1021/es1019614 PMC3031182

[10] Cui L, Zhou Q-f, Liao C-y, Fu J-j, Jiang G-b. Studies on the toxicological effects of pfoa and pfos on rats using histological observation and chemical analysis. Arch Environ Contam Toxicol 2009; 56:338–349. 10.1007/s00244-008-9194-6 18661093

[11] Dong G-H, Zhang Y-H, Zheng L, Liu W, Jin Y-H, He Q-C. Chronic effects of perfluorooctanesulfonate exposure on immunotoxicity in adult male c57bl/6 mice. Arch toxicol 2009; 83:805–815. 10.1007/s00204-009-0424-0 19343326

[12] Peden-Adams MM, EuDaly JG, Dabra S, EuDaly A, Heesemann L, Smythe J, et al Suppression of humoral immunity following exposure to the perfluorinated insecticide sulfluramid. J Toxicol & Environ Health, 2007 Part A 70:1130–1141. 10.1080/15287390701252733 17558808

[13] Peden-Adams MM, Keller JM, EuDaly JG, Berger J, Gilkeson GS, Keil DE. Suppression of humoral immunity in mice following exposure to perfluorooctane sulfonate. Toxicoll Sci 2008;104:144–154. 10.1093/toxsci/kfn059 18359764

[14] Fair PA, Driscoll E, Mollenhauer MAM, Bradshaw SG, Yun SH, Kannan K, et al Effects of environmentally-relevant levels of perfluorooctane sulfonate on clinical parameters and immunological functions in B_6_C_3_F_1_ mice. J Immunotoxicol 2011;8:Iss. 1. 10.3109/1547691X.2010.527868 PMC3446244 21261439

[15] Brieger A, Bienefeld N, Hasan R, Goerlich R, Haase H. 2011 Impact of perfluorooctanesulfonate and perfluorooctanoic acid on human peripheral leukocytes. Toxicol in Vitro 25:960–968. 10.1016/j.tiv.2011.03.005 21397682

[16] Corsini E, Avogadro A, Galbiati V, Marinovich M, Galli CL, Germolec DR. In vitro evaluation of the immunotoxic potential of perfluorinated compounds (pfcs). Toxicol Appl Pharmacol 2011;250:108–116. 10.1016/j.taap.2010.11.004 21075133

[17] Grandjean P, Andersen EW, Budtz-Jørgensen E, Nielsen F, Mølbak K, Weihe P, et al Serum vaccine antibody concentrations in children exposed to perfluorinated compounds. JAMA: 2012;307:391–397. 10.1001/jama.2011.2034 22274686 PMC4402650

[18] Granum B, Haug LS, Namork E, Stølevik SB, Thomsen C, Aaberge IS, et al Pre-natal exposure to perfluoroalkyl substances may be associated with altered vaccine antibody levels and immune-related health outcomes in early childhood. J Immunotox 2013;10:373–379. 10.3109/1547691X.2012.755580 23350954

[19] Looker C, Luster MI, Calafat AM, Johnson VJ, Burleson GR, Burleson FG, et al 2014. Influenza vaccine response in adults exposed to perfluorooctanoate and perfluorooctanesulfonate. Toxicol Sci 2014;138:76–88. 10.1093/toxsci/kft269 24284791 PMC4724206

[20] DeWitt JC, Shnyra A, Badr MZ, Loveless SE, Hoban D, Frame SR, et al Immunotoxicity of perfluorooctanoic acid and perfluorooctane sulfonate and the role of peroxisome proliferator-activated receptor alpha. Critical Rev Toxicol 2009;39:76–94. 18802816 10.1080/10408440802209804

[21] Watson JC, Hadler SC, Dykewicz CA, Reef S, Phillips L. 1998. Measles, mumps, and rubella-vaccine use and strategies for elimination of measles, rubella, and congenital rubella syndrome and control of mumps: Recommendations of the Advisory Committee on Immunization Practices (acip). 1998;47 (no. Rr-8.DTIC) Document. 9639369

[22] Harda K, Inue K, Morikawa A, Yoshinaga T, Saito N, Koizumi a. Renal clearance of perfluorooctane sulfonate and perfulorooctanoate in humans and their species-specific excretion. Environ Res 2005;99:253–361. 10.1016/j.envres.2004.12.003 16194675

[23] Lorber M, Eaglesham GE, Hobson P, Toms L-ML, Mueller JF, Thompson JS. The effect of ongoing blood loss on human serum concenteraations of perfluorinated acids. Chemosphere 2015;118:170–177. 10.1016/j.chemosphere.2014.07.093 25180653

[24] Li Y, Mucs D, Scott K, Lindh C, Tallving P, Fletcher T, Technical Report: No 2:2017. Half-lives of PFOS, PFHxS and PFOA after end of exposure to contaminated drinking water. The Sahlgrenska Academy Institute of Medicine, Gӧteborgs Universitet. 10.1136/oemed-2017-104651 PMC5749314 29133598

[25] Wong F, MacLeod M, Mueller JF, Cousins IT. Enhanced elimination of perfluorooctane sulfonic acid by menstruating women: Evidence from population-based pharmacokinetic modeling. Environ Sci Technol 2014, 48:8807–8814. 10.1021/es500796y 24943117

[26] Verner M-A, Longnecker MP. Comment on “Enhanced elimination of perfluorooctanesulfonic acid by menstruating women: Evidence from popoulatioin-based pharmacokinetic modeling. Environ Sci Technol 2015;49:5836–5837. 10.1021/acs.est.5b00187 25871968

[27] Centers for Disease Control and Prevention. 2014. National health and nutrition examination survey. http://www.cdc.gov/nchs/nhanes/about_nhanes.htm.

[28] Skendzel LP. Rubella immunity. Defining the level of protective antibody. Am J Clin Pathol 1996;106:170–174. 8712168 10.1093/ajcp/106.2.170

[29] Cook IF. Sexual dimorphism of humoral immunity with human vaccines. Vaccine 2008;26:3551–3555. 10.1016/j.vaccine.2008.04.054 18524433

[30] Buckley C, Dorsey F. Serum immunoglobulin levels throughout the life-span of healthy men. Ann Inter Med 1971;75:673–682. 10.7326/0003-4819-75-5-673 5122154

[31] Knox SS, Jackson T, Javins B, Frisbee SJ, Shankar A, Ducatman AM. Implications of early menopause in women exposed to perfluorocarbons. J Clin Endocrin & Metabol 2011; 96:1747–1753. 10.1210/jc.2010-2401 PMC3206400 21411548

[32] Stein CR, McGovern KJ, Pajak AM, Magilione PJ, Wolff MS. Perfluoroalkyl and polyfluoroalkyl sutstances and indicatoars of immune function in children aged 12–19 years: NHANES. Pediatr Res 2016;79:348–357. 26492286 10.1038/pr.2015.213 PMC5065061

[33] Chalubinski M, Kowalski ML. Endocrine disrupters–potential modulators of the immune system and allergic response. Allergy 2006;61:1326–1335. 10.1111/j.1398-9995.2006.01135.x 17002710

[34] Milla S, Depiereux S, Kestemont P. the effects of estrogenic and androgenic endocrine disrupters on the immune system of fish: a review. Ecotoxicology 2011;20:305–319. 10.1007/s10646-010-0588-7 21210218

[35] Jin Y, Chen R, Liu W, Fu Z. Effect of endocrine disrupting chemicals on the transcription of genes related to the innate immune sytem in the early developmental stage of zebrafish (Danio rerio). Fish & Shellfish Immun 2010;10:854–861. 10.1016/j.fsi.2010.02.009 20153439

[36] Dhiman N, Ovsyannikova IG, Vierkant RA, Pankratz V, Jacobson RM, Poland GA. Associations between cytokine/cytokine receptor single nucleotide polymorphisms and humoral immunity to measles, mumps and rubella in a somali population. Tissue Antigens 2008;72:211–220. 10.1111/j.1399-0039.2008.01097.x 18715339 PMC2595143

[37] Ovsyannikova IG, Jacobson RM, Dhiman N, Vierkant RA, Pankratz VS, Poland GA. Human leukocyte antigen and cytokine receptor gene polymorphisms associated with heterogeneous immune responses to mumps viral vaccine. Pediatrics 2008 121:e1091–e1099. 10.1542/peds.2007-1575 18450852 PMC2668976

[38] Kennedy RB, Ovsyannikova IG, Pankratz VS, Vierkant RA, Jacobson RM, Ryan MA, et al Gender effects on humoral immune responses to smallpox vaccine. Vaccine 2009;27:3319–3323. 10.1016/j.vaccine.2009.01.086 19200827 PMC2831636

[39] Engler RJ, Nelson MR, Klote MM, VanRaden MJ, Huang C-Y, Cox NJ, et al Half-vs full-dose trivalent inactivated influenza vaccine (2004–2005): Age, dose, and sex effects on immune responses. Arch Inter Med 2008;168:2405. 10.1001/archinternmed.2008.513 19064822

[40] Mitchell LA, Zhang T, Tingle AJ. *The Journal of Infectious Diseases* 1992;166:1258–1265. 1431244 10.1093/infdis/166.6.1258

[41] Cooper GS, Stroehla BC. The epidemiology of autoimmune diseases. *Autoimmunity Reviews* 2003;2:119–125. 12848952 10.1016/s1568-9972(03)00006-5

